# Blocking the janus-activated kinase pathway reduces tumor necrosis factor alpha-induced interleukin-18 bioactivity by caspase-1 inhibition

**DOI:** 10.1186/ar4551

**Published:** 2014-04-24

**Authors:** Hubert Marotte, Pei-Suen Tsou, Tatiana Fedorova, Adam J Pinney, Benjamin Lewis, Alisa E Koch

**Affiliations:** 1 Department of Internal Medicine/Division of Rheumatology, University of Michigan Medical School, Ann Arbor, MI 48109, USA; 2 Currently INSERM U1059 Laboratoire Biologie intégrée du Tissu Osseux, Université de Lyon, F-42023 Saint-Etienne, France; 3 VA Medical Service, Department of Veterans Affairs Medical Center, Ann Arbor, MI 48108, USA

## Abstract

**Introduction:**

Our objective was to examine the role of the janus-activated kinase (JAK) pathway in the modulation of tumor necrosis factor-α (TNF)-induced-IL-18 bioactivity by reduction of caspase-1 function.

**Methods:**

Caspase-1 expression in rheumatoid arthritis (RA) synovial fibroblasts treated with TNF was assessed by qRT-PCR and Western blot. Interleukin (IL)-18 was assessed by enzyme-linked immunosorbent assay (ELISA) in cell lysates and conditioned media and detected by immunofluorescence (IF) staining in RA synovial fibroblasts. The critical pathways for TNF-induced caspase-1 expression were determined by using chemical inhibitors of signaling followed by TNF stimulation. IL-18 bioactivity was assessed using human myelomonocytic KG-1 cells.

**Results:**

TNF induced RA synovial fibroblast caspase-1 expression at the protein level in a time-dependant manner (*P* < 0.05). Blocking the JAK pathway reduced TNF-induced-caspase-1 expression at the transcriptional and protein levels by approximately 60% and 40%, respectively (*P* < 0.05). Blocking the JAK pathway reduced TNF-induced-caspase-1 expression at the transcriptional, protein, and activity levels by approximately 60%, 40%, and 53%, respectively (*P* < 0.05). We then confirmed by IF that TNF-induced IL-18 and investigated roles of the ERK1/2 and JAK pathways. Blocking the JAK pathway, TNF induced intracytoplasmic granular IL-18 expression suggesting a defect of caspase-1. Finally, blocking the JAK pathway, we observed a reduction of IL-18 bioactivity by 52% in RA synovial fibroblasts (*P* < 0.05).

**Conclusions:**

These results provide a new way to regulate TNF-induced-IL-18 bioactivity by blocking capase-1. These data present a novel role for JAK inhibition in RA patients and emphasize JAK inhibition use as a new therapeutic option in RA management.

## Introduction

Rheumatoid arthritis (RA) is characterized by inflamed synovial tissue containing a massive infiltration of lymphocytes and macrophages with synovial fibroblast proliferation. IL-18, an IL-1 family member, is involved in RA pathogenesis [1]. We and others have shown that IL-18 plays an important role in the immune response, in local or systemic angiogenesis [2,3], and in monocyte recruitment [4]. Various sources of IL-18 have been identified including antigen-presenting cells, as well as keratinocytes, articular chondrocytes, osteoblasts, and synovial fibroblasts [5,6].

IL-18, is produced as a biologically inactive precursor protein (pro-IL-18) containing a propeptide domain localized to the cytoplasm. To be activated, pro-IL-18 requires cleavage by the IL-1β-converting enzyme (ICE), which is a member of the aspartate-specific cysteine protease family (caspase-1). Caspase-1 is produced as an inactive form. To be activated, its needs to be cleaved into 20 kDa and 10 kDa subunits. Both subunits form heterodimers with interactions with other proteins and are involved in inflammasome formation and activation of inflammatory processes [7]. Active caspase-1 is located in the plasma membrane, where it cleaves pro-IL-18 (inactive) to IL-18 (still inactive). Caspase-1 and pro-IL-18/IL-18 are complexed to other proteins that are involved in the secretion of IL-18 [8,9]. Caspase-1 is also a critical putative target in patients with cryopyrin-associated periodic syndromes (CAPS) [10]. When IL-18 is secreted, it becomes active [11]. IL-18 bioactivity is dependent on both IL-18 and IL-18 binding protein (IL-18BP, its natural inhibitor) levels [5,6].

Among various signaling pathways, the mitogen-activated protein kinase (MAPK) family, nuclear factor kappa-light-chain-enhancer of activated B cells (NF-κB) and janus-activated kinase (JAK) pathways are thought to be critical in RA pathogenesis [12]. All these pathways can be activated by TNF-α [5,6]. We previously described ways to regulate TNF-induced IL-18 bioactivity in RA synovial fibroblasts by modulation of IL-18 or IL-1BP [5,6]. Here, we explore regulation of TNF-induced IL-18 bioactivity by reduction of TNF-induced caspase-1 in RA synovial fibroblasts.

## Methods

### Cytokines, culture of human RA synovial fibroblasts, and chemical inhibitors

TNF was purchased from R&D Systems (Minneapolis, MN, USA). Fibroblasts were isolated from RA synovium obtained from RA patients undergoing arthroplasty or synovectomy as described previously [5,6]. For all human specimens used in this study, we obtained written informed consent and all aspects of the study were approved by the University of Michigan Institutional Review Board. Allergies were not reported and no skin tests were performed on these RA patients. MAPK inhibitors (ERK1/2, PD98059; p38, SB202190; and JNK2, SP600125), an NF-κB inhibitor (pyrrolidine dithiocarbamate (PDTC)), or a JAK2 inhibitor (AG490) were used at 10 μM of each inhibitor, except PDTC at 200 μM [5,6]. All inhibitors were purchased from Calbiochem (San Diego, CA, USA). All experiments were performed in serum-free media except experiments for IL-18 detection.

### Cell lysis and western blotting

To study the effect of TNF on caspase-1 expression, RA synovial fibroblasts were incubated with TNF (20 ng/ml) in RPMI 1640 and processed, as previously described [5,6]. We used a rabbit anti-human caspase-1 antibody (Santa Cruz Biotechnology, Santa Cruz, CA, USA) overnight at 4°C and then horseradish peroxidase-conjugated antibody (1:1,000 dilution) for 1 hour at room temperature. Blots were scanned and analyzed for band intensities, as previously described [5,6].

### Caspase-1 activity assay

RA synovial fibroblasts (2 × 10^6^/well) were pre-incubated with the chemical inhibitors (described above) for 2 hours and then treated with TNF (20 ng/ml) for 24 hours in serum-free RPMI 1640. Cells were washed and then lysed with the lysis buffer from the caspase-1 activity assay kit. Cell lysates were centrifuged, and the supernatant was assessed. Caspase-1 activity in the supernatant was determined using a colorimetric caspase-1 activity assay kit (R&D Systems).

### IL-18 detection in conditioned media

RA synovial fibroblasts were stimulated with TNF (20 ng/ml) in RPMI 1640 with 10% fetal bovine serum (FBS) supplementation for 72 hours. Conditioned medium was collected and concentrated 10-fold using Amicon Ultra 3,000 MW concentrators from Millipore (Bedford, MA, USA). Equal volumes of conditioned media were loaded and processed for western blotting as previously described above except that primary polyclonal rabbit anti-human IL-18 antibody was used (Santa Cruz biotechnology, Santa Cruz, CA, USA).

### ELISA for IL-18 and IL-18BP

RA synovial fibroblasts were stimulated with TNF for 8 to 48 hours in RPMI with 10% FBS and conditioned medium was collected and concentrated as described above. IL-18 was assessed in conditioned media and cell lysates using an ELISA kit from Bender MedSystems (Burlingame, CA, USA). IL-18BP was assessed in conditioned media using an ELISA kit from R&D Systems.

### RNA extraction and quantitative real time-polymerase chain reaction (qRT-PCR)

Following the manufacturer’s protocol, RNA was isolated from RA synovial fibroblasts and processed as described previously [5,6]. We used specific primer sequences for caspase-1: forward: 5′-AGCCAACATGCCCATCACTCGG-3′, reverse: 5′- TGCTACGGTGCACAGGGAATGG -3′; and for β-actin: forward: 5′-GTCAGGCAGCTCGTAGCTCT-3′, reverse: 5′-GCCATGTACGTTGCTATCCA-3′ and the following cycles: 50°C for 2 minutes, 95°C for 2 minutes, and 40 cycles of 95°C for 30 seconds, 55°C for 30 seconds, and 68°C for 30 seconds. For quantification, the relative abundance of each gene was normalized to β-actin.

### IL-18 bioactivity

The biologic activity of IL-18 was measured using human myelomonocytic KG-1 cells, as previously described [5,6]. KG-1 cells (3 × 10^6^ cells/ml; 100 μL), with or without mouse monoclonal anti-IL-18 antibody at 1 μg/ml (R&D Systems), were dispensed into the wells of 96-well Falcon microtiter plates (Becton Dickinson). Next, 100 μL of samples or recombinant human IL-18 standard was added to each well. The plates were incubated, and culture supernatants were harvested 24 hours later. The IFNγ concentration in this media was determined by ELISA (Invitrogen, Grand Island, NY, USA). IL-18 bioactivity was determined based on the difference in IFNγ levels between cultures with and those without mouse anti-IL-18 monoclonal antibody.

### Immunofluorescence staining

RA synovial fibroblasts were plated in 8-well Labtek chamber slides and processed as described previously [6]. Briefly, cells were untreated or stimulated with TNF (20 ng/ml) for 48 hours with or without preincubation with PD98059 or AG490 for 2 hours. After 48 hours, cells were washed, fixed, permeabilized, and blocked. IL-18 primary antibody (10 μg/ml), which reacts with both immature and mature IL-18 forms, was used after washing in combination with Alexa Fluor-conjugated goat anti-rabbit antibody. After washing, nuclei were stained with 4′,6′-diamidino-2-phenylindole (Invitrogen). Slides were dehydrated, mounted, and coverslipped. Immunofluorescence (IF) staining was detected using an Olympus FV-500 microscope (Olympus America, Melville, NY, USA).

### Statistical analysis

Statistically significant differences between groups were calculated using Student’s *t*-test. *P*-values less than 0.05 were considered significant. All statistical data are expressed as the mean ± standard error of the mean (SEM).

## Results

### TNF induced functional caspase-1 in RA synovial fibroblasts

**Figure 1:**
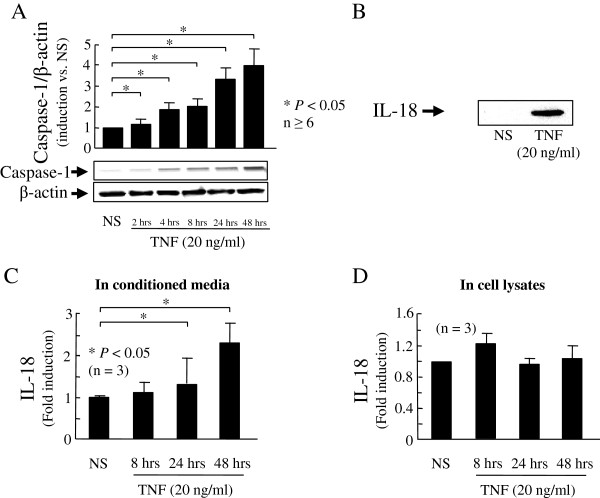
**TNF-induced functional caspase-1 in rheumatoid arthritis (RA) synovial fibroblasts.** RA synovial fibroblasts (2 × 10^5^/well plates; 2 ml/well) were stimulated with TNF in serum-free media for 0 to 48 hours. Caspase-1 expression was assessed at the protein level in cell lysates. Bands shown are specific for active caspase-1. Bars show the mean and standard error of the mean (SEM) from at least six independent experiments using different donors **(A)**. RA synovial fibroblasts (2 × 10^5^/well in 6-well plates; 2 ml/well) were stimulated in RPMI with 10% FBS for 72 hours. IL-18 was detected by western blotting in 10-times concentrated conditioned media. A representative blot of three independent experiments using different donors is shown **(B)**. RA synovial fibroblasts (2 × 10^5^/well in 6-well plates; 2 ml/well) were treated with TNF (20 ng/ml) for 8, 24, and 48 hours in RPMI with 10% FBS. IL-18 protein induction was assessed by ELISA in concentrated conditioned media **(C)** and cell lysates **(D)**. Results are expressed as the mean of fold induction with SEM. *P <0.05 versus non stimulated (NS).

To determine whether pro-IL-18 was potentially cleaved by active caspase-1 to the IL-18 active form, we examined caspase-1 expression in cell lysates and IL-18 expression in cell lysates and conditioned media at the protein level, without or with TNF stimulation. TNF induced caspase-1 at the protein level in cell lysates in a time-dependent manner (Figure 1A) and the mature IL-18 secretion in the conditioned media assessed by western blot and ELISA (Figure 1B,C). The pro-IL-18 level in cell lysates did not change over time (Figure 1D), suggesting that pro-IL-18 is cleaved to IL-18 and then secreted. These data indicate that TNF induced functional caspase-1 to cleave pro-IL-18.

### Role of the JAK pathway in TNF-induced caspase-1

**Figure 2:**
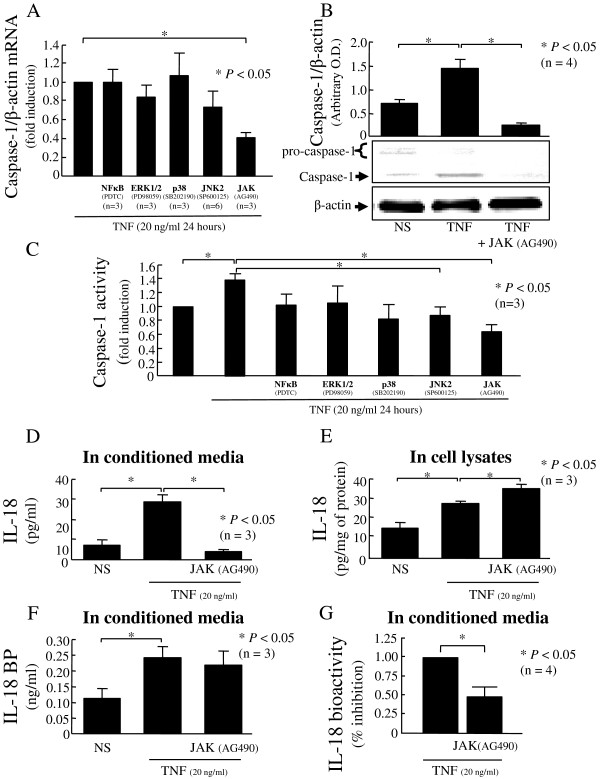
**The JAK pathway: a critical pathway for TNF-induced functional caspase-1.** Rheumatoid arthritis (RA) synovial fibroblasts (2 × 10^5^/well; 2 ml/well) were pre-incubated with the indicated inhibitors for 2 hours, followed by stimulation with TNF (20 ng/ml) for 48 hours. Caspase-1 was assessed at the mRNA level **(A)**, the protein level **(B)**, and its activity was measured **(C)**. RA synovial fibroblasts (2 × 10^5^/well; 2 ml/well; in RPMI with 10% fetal bovine serum (FBS)) were pre-incubated with AG490 for 2 hours, followed by stimulation with TNF for 48 hours. Medium was collected and concentrated, while cell lysates were processed. IL-18 levels were assessed by ELISA in both conditioned media **(D)** and cell lysates **(E)**. IL-18BP levels **(F)** and IL-18 bioactivity **(G)** were assessed in the conditioned media. Bars show the mean ± standard error of the mean (SEM). *P <0.05. NS, non stimulated; n, number of donors and independent experiments.

To identify signaling events that are critical for TNF-induced caspase-1, RA synovial fibroblasts were incubated with chemical signaling inhibitors for 2 hours, followed by TNF stimulation. Only JAK pathway inhibition significantly decreased TNF-induced caspase-1 at the transcriptional level in RA synovial fibroblasts (57% reduction; *P* <0.05; n = 3; Figure 2A). TNF-induced caspase-1 protein expression was markedly reduced when the JAK pathway was blocked in RA synovial fibroblasts (*P* <0.05; n = 4; Figure 2B). According to our blot, this reduction is due mainly to a reduction of pro-caspase-1 expression. At the end, we assessed the functional activity of capsase-1. Blocking the JAK pathway strongly reduced TNF-induced caspase-1 activity (*P* <0.05; n = 3; Figure 2C). Furthermore, blocking the JNK pathway already slightly decreased the TNF-induced caspase-1 activity (*P* <0.05; n = 3; Figure 2C). These data indicate that the JAK pathway is a critical pathway for TNF-induced caspase-1 and IL-18 bioactivity.

### Blocking JAK results in reduction of TNF-induced IL-18 bioactivity in RA synovial fibroblasts

After showing the key role of JAK in TNF-induced caspase-1 expression and activity, we assessed its function on maturation of IL-18. In conditioned media, JAK blockade potently decreased TNF-induced IL-18 (*P* < 0.05; n = 4; Figure 2D), whereas IL-18BP was not affected (Figure 2F). In cell lysates, when JAK was blocked, TNF-induced IL-18 increased, suggesting a defect of IL-18 secretion (*P* <0.05; n = 4; Figure 2E). As IL-18 bioactivity is the result of the balance between mature secreted IL-18 and IL-18BP, we explored IL-18 bioactivity in the same conditioned media using KG-1 cells. We confirmed that TNF induced IL-18 bioactivity and this induction was reduced by 52% after blockade of the JAK pathway (*P* <0.05; n = 4; Figure 2G). The data confirmed that blocking the JAK pathway reduced IL-18 bioactivity without effect on IL-18BP.

### Blocking caspase-1 results in inhibition of release of IL-18

**Figure 3:**
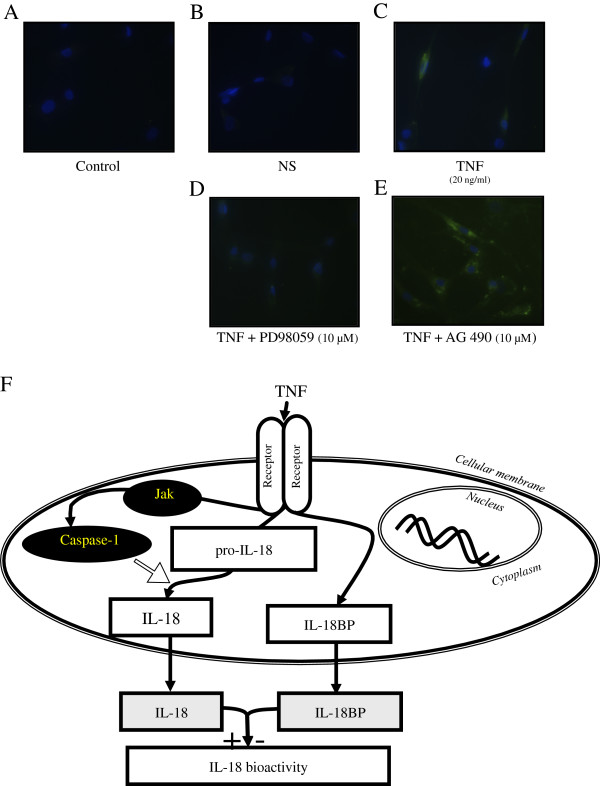
**Localization of IL-18 on rheumatoid arthritis (RA) synovial fibroblasts with or without stimulation with TNF (20 ng/ml for 2 hours) was examined by immunofluorescence.** RA synovial fibroblasts were pre-incubated for 2 hours with PD98059 or AG490 (ERK1/2 and JAK pathway chemical inhibitors at 10 μM, respectively) and then stimulated with TNF (20 ng/ml) for 48 hours. Control IgG **(A)** and IL-18 **(B)** detection without stimulation showed no staining. After 48 hours of TNF stimulation, we observed staining in the cytoplasm **(C)**. Upon TNF stimulation, after blocking the ERK1/2 pathway, no detection was observed in the cytoplasm **(D)**. Upon TNF stimulation, after blocking the JAK pathway, IL-18 was detected in the cytoplasm with some granularity **(E)**. Images shown are representative of three independent experiments. Schematic representation of TNF induction of mature IL-18 by induction of functional caspase-1 through the JAK pathway **(F)**.

IL-18 expression inside the cell was detected using IF in various stimulation conditions. We confirmed induction of expression of pro-IL-18 by TNF (Figure 3C). To validate this assay, we blocked the ERK pathway, which was previously reported to be critical for TNF-induced-pro-IL-18 and observed inhibition of IL-18 after TNF stimulation (Figure 3D) [5]. Additionally upon blocking JAK, we observed an intracytoplasmic granular staining (Figure 3E). This suggests accumulation of pro-IL-18 without secretion, suggesting a lack of effect of caspase-1. These results indicate a crucial role of the JAK pathway in regulating TNF-induced IL-18 bioactivity (Figure 3F). The data confirmed that blocking the JAK pathway reduced IL-18 bioactivity by IL-18 maturation reduction.

## Discussion

Compared to other pro-inflammatory cytokines, IL-18 is highly regulated at the expression, maturation, and bioactivity levels. Constitutive IL-18 mRNA and protein in the precursor form are present in non stimulated human cells and in untreated tissues [13]. Without stimulation, IL-18 is primarily present in the precursor form, which requires conversion by caspase-1 to the mature and bioactive form [11]. The membrane-bound form of IL-18 was recently described to be caspase-1 dependent and restricted to a subgroup of monocytes [14].

Here, we confirmed that TNF induced caspase-1 in a time-dependent manner at both protein and activity levels in RA synovial fibroblasts, as previously suggested [5]. We also confirmed that TNF induced IL-18 expression and secretion from RA synovial fibroblasts [5]. IL-18 in the conditioned media after TNF induction suggested the presence of functional TNF-induced caspase-1. This is consistent with previous data showing that TNF induces IL-1β [15].

AG490 is mainly a strong inhibitor of JAK2. However, it was described to also inhibit the JAK3 pathway. Hence, these inhibitors are not specific enough to claim JAK2 specificity. We previously described that the JAK pathway was not involved in TNF-induced IL-18 or IL-18BP in the same *in vitro* model [5]. As a result, in this model of IL-18 bioactivity induced by TNF, we describe a new way to reduce IL-18 bioactivity by regulation of caspase-1. Previously, we observed that the ERK pathway was critical for IL-18 expression [5], whereas the JNK-2 and NF-κB pathways were important for IL-18BP expression [6]. Compared to our previous results, we found a new specific pathway for regulating IL-18 bioactivity, that is, the JAK pathway.

TNF induces many intracellular signaling pathways. The JAK pathway is activated by TNF through its binding to its type I receptor [16]. Furthermore, expression of chemokines induced by TNF was reduced by blocking the JAK pathway in RA synovial fibroblasts [17] and in RA synovial macrophages [18]. So in this model, blocking the JAK2 pathway specifically reduced TNF-induced IL-18 bioactivity only by reduction of IL-18 secretion due to inhibition of functional caspase-1. *In vivo*, JAK2 pathway inhibition has been shown to improve inflammatory arthritis in a rodent model [19] and blocking JAK1/3 has been shown to reduce joint destruction [20]. JAK inhibitors suppress both innate and adaptative immunity in the K/BxN serum transfer model [18]. In human RA, JAK inhibitors are a new attractive therapeutic option for RA management [21].

## Conclusions

These results provide a novel way to regulate TNF-induced-IL-18 bioactivity by blocking capase-1. These results suggest an additional mechanism to explain the beneficial effect of JAK inhibitors in RA.

## Abbreviations

CAPS: cryopyrin-associated periodic syndromes; FBS: fetal bovine serum; ICE: interleukin-1β-converting enzyme; IF: immunofluorescence; IFNγ: interferon-γ; IL: interleukin; JAK: Janus-activated kinase; MAPK: mitogen-activated protein kinase; NF-κB: nuclear factor kappa-light-chain-enhancer of activated B cells; PDTC: pyrrolidine dithiocarbamate; RA: rheumatoid arthritis; TNF: tumor necrosis factor.

## Competing interests

The authors except Dr Koch have no competing interests. Dr Koch recently joined Eli Lilly Pharmaceutical Company. However, the work was performed prior to that.

## Authors’ contributions

HM: conception and design, data collection and analysis, manuscript writing and final approval of the manuscript. PST: data collection and analysis, critical revision and final approval of the manuscript. TF: data collection and analysis, critical revision and final approval of the manuscript. AJP: data collection and analysis, critical revision and final approval of the manuscript. BL: data collection and analysis, critical revision and final approval of the manuscript. AEK: data collection and analysis, financial support, critical revision and final approval of the manuscript. All authors read and approved the final manuscript.

## References

[B1] Gracie JA Forsey RJ Chan WL Gilmour A Leung BP Greer MR Kennedy K Carter R Wei XQ Xu D Field M Foulis A Liew FY McInnes IB A proinflammatory role for IL-18 in rheumatoid arthritis J Clin Invest 1999 104 1393 1401 10.1172/JCI7317 10562301 PMC409841

[B2] Morel JC Park CC Zhu K Kumar P Ruth JH Koch AE Signal transduction pathways involved in rheumatoid arthritis synovial fibroblast interleukin-18-induced vascular cell adhesion molecule-1 expression J Biol Chem 2002 277 34679 34691 10.1074/jbc.M206337200 12105209

[B3] Amin MA Rabquer BJ Mansfield PJ Ruth JH Marotte H Haas CS Reamer EN Koch AE Interleukin 18 induces angiogenesis in vitro and in vivo via Src and Jnk kinases Ann Rheum Dis 2010 69 2204 2212 10.1136/ard.2009.127241 20679476

[B4] Ruth JH Park CC Amin MA Lesch C Marotte H Shahrara S Koch AE Interleukin-18 as an in vivo mediator of monocyte recruitment in rodent models of rheumatoid arthritis Arthritis Res Ther 2010 12 R118 10.1186/ar3055 20565717 PMC2911912

[B5] Marotte H Ahmed S Ruth JH Koch AE Blocking ERK-1/2 reduces tumor necrosis factor alpha-induced interleukin-18 bioactivity in rheumatoid arthritis synovial fibroblasts by induction of interleukin-18 binding protein A Arthritis Rheum 2010 62 722 731 10.1002/art.27269 20131228 PMC2855552

[B6] Marotte H Tsou PS Rabquer BJ Pinney AJ Fedorova T Lalwani N Koch AE Blocking of interferon regulatory factor 1 reduces tumor necrosis factor alpha-induced interleukin-18 bioactivity in rheumatoid arthritis synovial fibroblasts by induction of interleukin-18 binding protein a: Role of the nuclear interferon regulatory factor 1-NF-kappaB-c-jun complex Arthritis Rheum 2011 63 3253 3262 10.1002/art.30583 21834067 PMC4416229

[B7] Fink SL Cookson BT Caspase-1-dependent pore formation during pyroptosis leads to osmotic lysis of infected host macrophages Cell Microbiol 2006 8 1812 1825 10.1111/j.1462-5822.2006.00751.x 16824040

[B8] Martinon F Tschopp J Inflammatory caspases: linking an intracellular innate immune system to autoinflammatory diseases Cell 2004 117 561 574 10.1016/j.cell.2004.05.004 15163405

[B9] Franchi L Eigenbrod T Munoz-Planillo R Nunez G The inflammasome: a caspase-1-activation platform that regulates immune responses and disease pathogenesis Nat Immunol 2009 10 241 247 19221555 10.1038/ni.1703 PMC2820724

[B10] Dinarello CA Simon A van der Meer JW Treating inflammation by blocking interleukin-1 in a broad spectrum of diseases Nat Rev Drug Discov 2012 11 633 652 10.1038/nrd3800 22850787 PMC3644509

[B11] Gu Y Kuida K Tsutsui H Ku G Hsiao K Fleming MA Hayashi N Higashino K Okamura H Nakanishi K Kurimoto M Tanimoto T Flavell RA Sato V Harding MW Livingston DJ Su MS Activation of interferon-gamma inducing factor mediated by interleukin-1 beta converting enzyme Science 1997 275 206 209 10.1126/science.275.5297.206 8999548

[B12] Malemud CJ Miller AH Pro-inflammatory cytokine-induced SAPK/MAPK and JAK/STAT in rheumatoid arthritis and the new anti-depression drugs Expert Opin Ther Targets 2008 12 171 183 10.1517/14728222.12.2.171 18208366

[B13] Puren AJ Fantuzzi G Dinarello CA Gene expression, synthesis, and secretion of interleukin 18 and interleukin 1beta are differentially regulated in human blood mononuclear cells and mouse spleen cells Proc Natl Acad Sci USA 1999 96 2256 2261 10.1073/pnas.96.5.2256 10051628 PMC26770

[B14] Bellora F Castriconi R Doni A Cantoni C Moretta L Mantovani A Moretta A Bottino C M-CSF induces the expression of a membrane-bound form of IL-18 in a subset of human monocytes differentiating in vitro toward macrophages Eur J Immunol 2012 42 1618 1626 10.1002/eji.201142173 22678914

[B15] Dinarello CA Cannon JG Wolff SM Bernheim HA Beutler B Cerami A Figari IS Palladino MA Jr O’Connor JV Tumor necrosis factor (cachectin) is an endogenous pyrogen and induces production of interleukin 1 J Exp Med 1986 163 1433 1450 10.1084/jem.163.6.1433 3486936 PMC2188124

[B16] Guo D Dunbar JD Yang CH Pfeffer LM Donner DB Induction of Jak/STAT signaling by activation of the type 1 TNF receptor J Immunol 1998 160 2742 2750 9510175

[B17] Rosengren S Corr M Firestein GS Boyle DL The JAK inhibitor CP-690,550 (tofacitinib) inhibits TNF-induced chemokine expression in fibroblast-like synoviocytes: autocrine role of type I interferon Ann Rheum Dis 2012 71 440 447 10.1136/ard.2011.150284 22121136

[B18] Yarilina A Xu K Chan C Ivashkiv LB Regulation of inflammatory responses in tumor necrosis factor-activated and rheumatoid arthritis synovial macrophages by JAK inhibitors Arthritis Rheum 2012 64 3856 3866 10.1002/art.37691 22941906 PMC3510320

[B19] Fridman JS Scherle PA Collins R Burn TC Li Y Li J Covington MB Thomas B Collier P Favata MF Wen X Shi J McGee R Haley PJ Shepard S Rodgers JD Yeleswaram S Hollis G Newton RC Metcalf B Friedman SM Vaddi K Selective inhibition of JAK1 and JAK2 is efficacious in rodent models of arthritis: preclinical characterization of INCB028050 J Immunol 2010 184 5298 5307 10.4049/jimmunol.0902819 20363976

[B20] LaBranche TP Jesson MI Radi ZA Storer CE Guzova JA Bonar SL Thompson JM Happa FA Stewart ZS Zhan Y Bollinger CS Bansal PN Wellen JW Wilkie DP Bailey SA Symanowicz PT Hegen M Head RD Kishore N Mbalaviele G Meyer DM JAK inhibition with tofacitinib suppresses arthritic joint structural damage through decreased RANKL production Arthritis Rheum 2012 64 3531 3542 10.1002/art.34649 22899318

[B21] Fleischmann R Cutolo M Genovese MC Lee EB Kanik KS Sadis S Connell CA Gruben D Krishnaswami S Wallenstein G Wilkinson BE Zwillich SH Phase IIb dose-ranging study of the oral JAK inhibitor tofacitinib (CP-690,550) or adalimumab monotherapy versus placebo in patients with active rheumatoid arthritis with an inadequate response to disease-modifying antirheumatic drugs Arthritis Rheum 2012 64 617 629 10.1002/art.33383 21952978

